# EATING EXPERIENCES IN PEOPLE LIVING WITH DEMENTIA: A CONCEPT ANALYSIS USING RODGERS’S METHODOLOGY

**DOI:** 10.1093/geroni/igae098.3074

**Published:** 2024-12-31

**Authors:** Zih-Ling Wang, Jenna McHale, Basia Belza, Jennifer Sonney

**Affiliations:** University of Washington, Seattle, Washington, United States; University of Washington, Seattle, Washington, United States; University of Washington, Seattle, Washington, United States; University of Washington, Seattle, Washington, United States

## Abstract

In people living with dementia, eating has often been portrayed in the literature as a time-consuming process or task-centered activity. However, it is important to distinguish the experience of eating from the process and task-centered approach of feeding. This study clarifies the concept of eating experience by using Rodgers’ evolutionary approach to analyzing factors associated with eating experiences in people living with dementia present in the literature. Twenty-two articles met the inclusion criteria, identifying three key attributes of eating experiences (self-connection, the special journey of life, and self-interpretation). Antecedents, as framed by the socio-ecological model, were categorized to represent Intrapersonal (personal preferences, individual culture, mealtime routines), Interpersonal (social interaction), and Environmental (dining room environment, policies) factors. Consequences were divided into external (quality of life, nutritional health, and physical health) and internal (personhood, autonomy and independence, dignity and feeling valued, and mental well-being) domains. The identified attributes, antecedents, and consequences can be utilized in healthcare education, research, and intervention approaches. This presentation allows nurses and other health care professionals to understand better people living with dementia through the relationship between eating and interpersonal, intrapersonal, and environmental aspects to develop personalized interventions and care strategies to achieve an optimal quality of life.

